# Quantifying microscale drivers for fatigue failure via coupled synchrotron X-ray characterization and simulations

**DOI:** 10.1038/s41467-020-16894-2

**Published:** 2020-06-24

**Authors:** Sven Gustafson, Wolfgang Ludwig, Paul Shade, Diwakar Naragani, Darren Pagan, Phil Cook, Can Yildirim, Carsten Detlefs, Michael D. Sangid

**Affiliations:** 1 School of Aeronautics and Astronautics, Purdue University, 701W. Stadium Ave, West Lafayette, IN 47906 USA; 2 University Lyon I, MATEIS, UMR5510 CNRS, 25 av. J. Capelle, 69621 Villeurbanne, France; 3 European Synchrotron Radiation Facility, Beamline ID06, 71 Avenue des Martyrs, 38000 Grenoble, France; 4 Materials and Manufacturing Directorate, Air Force Research Laboratory, Wright-Patterson AFB, OH 45433 USA; 5 Cornell High Energy Synchrotron Source, Ithaca, NY USA; 6 Present Address: LETI, CEA, 17 Avenue des Martyrs, Grenoble, 38054 France

**Keywords:** Mechanical properties, Metals and alloys, Characterization and analytical techniques

## Abstract

During cyclic loading, localization of intragranular deformation due to crystallographic slip acts as a precursor for crack initiation, often at coherent twin boundaries. A suite of high-resolution synchrotron X-ray characterizations, coupled with a crystal plasticity simulation, was conducted on a polycrystalline nickel-based superalloy microstructure near a parent-twin boundary in order to understand the deformation localization behavior of this critical, 3D microstructural configuration. Dark-field X-ray microscopy was spatially linked to high energy X-ray diffraction microscopy and X-ray diffraction contrast tomography in order to quantify, with cutting-edge resolution, an intragranular misorientation and high elastic strain gradients near a twin boundary. These observations quantify the extreme sub-grain scale stress gradients present in polycrystalline microstructures, which often lead to fatigue failure.

## Abstract (web summary)

Structural alloys have distinct microstructural features known as twins that are preferential sites for fatigue crack initiation and need to be better understood to mitigate catastrophic failures. Here, the authors show unusually large stress gradients near a twin boundary, using X-ray techniques and modelling.

## Introduction

The use of polycrystalline metallic alloys forms the backbone of many industries influencing everyday life including infrastructure, power generation, and air transportation. Fatigue crack initiation, a predominant failure mechanism in many of the structural materials used in these applications, is currently mitigated by large-scale testing programs and conservative design practices. The primary downsides of these approaches are the necessity to perform costly, preventive repair and maintenance routines, and the premature removal of useful parts from service to ensure continued safe operation. Ultimately, these practices are required by the fact that a deterministic failure criterion for materials, at the microscale (where failure initiates), are not sufficiently understood, and while detailed modeling approaches have been employed, more work is necessary to validate the failure criterion. In an effort to minimize these routines and to improve predictive capabilities, researchers have sought a more detailed description and understanding of polycrystalline deformation and the rare deformation localization events that lead to crack initiation. This requires coupling specifically designed, multi-modal experiments with physics-based computational models, where the role of the experiment is to provide information of the underpinning physics and datasets for model validation.

Nickel-based superalloys are instrumental in high temperature structural applications. They are, however, known to form fatigue cracks near coherent twin boundaries (low energy configuration grain boundaries separating twin and parent grains) from surface characterization and fracture surface analysis^1–4^. Load applied to a crystal (a grain) will elastically stretch bonds between atoms and place the material in a state of stress. With sufficient applied load, the crystalline lattice will plastically deform, which, in the superalloys of interest, is primarily facilitated by the generation and motion of line defects (dislocations). Extensive localized plasticity can then occur due to combinations of local boundary conditions and dislocation activity, which eventually produce stress concentrations and subsequently, initiate a crack^5^. This localization often occurs at grain boundaries^6^, especially coherent twin boundaries, like those present in nickel-based superalloys. While the presence of twin boundaries adds strength and ductility to nickel-based superalloys, often increasing the fatigue performance^7^, they are also known to be a primary site for fatigue crack nucleation and failure^8–12^.

**Fig. 1:**
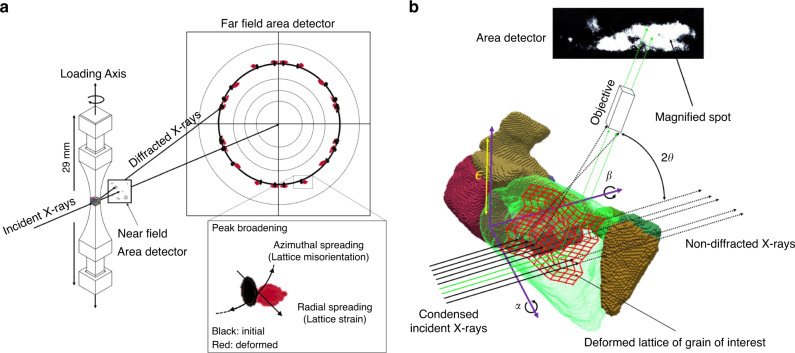
X-Ray Characterization Techniques. (**a**) Schematic of HEDM setup and example of spot deformation due to macroscopic loading. (**b**) Schematic of DFXM setup. X-rays colored in green are those which both diffract and pass through the objective.

To understand the physical phenomena occurring during plastic deformation and the subsequent crack initiation, recent advances have sought to use X-ray diffraction to probe the micromechanical response and microstructural evolution during macroscopic loading. High energy X-ray diffraction microscopy (HEDM) is an X-ray technique used to analyze the diffracted X-rays from individual grains of millimeter sized samples (Fig. 1a). Other techniques have been explored such as transmission electron microscopy (TEM) and high-resolution electron backscatter diffraction (HR-EBSD), but these either alter boundary conditions, allowing stresses to relax, or only characterize surface deformation^13,14^. Techniques such as diffraction contrast tomography (DCT) and HEDM can provide information on the initiation and growth mechanics of fatigue cracks; however, they currently cannot reconstruct intragranular elastic strain information^15,16^. Recent advances in X-ray diffraction experiments have allowed for the acquisition of misorientation and elastic strain via dark-field X-ray microscopy (DFXM), which probes a single set of lattice planes within a grain of interest (GOI) with high spatial and angular resolution^17–21^ (Fig. 1b). Other X-ray techniques, such as X-ray microdiffraction techniques, can provide similar, high resolution, information in similarly sized volumes^22–24^. However, there are several hurdles to overcome to register these spatially dependent X-ray techniques in order to create a set of robust results that include not just the grain morphological information, but also strain information at the sub-grain level.

Therefore, this work strategically designs and links multi-modal experiments, specifically utilizing DFXM characterization of the intragranular orientation and elastic strain near a coherent twin boundary of the GOI, in the context of the neighboring microstructure. This has allowed for an insightful investigation of the micromechanical rationale for strain localization as a precursor to crack initiation in a polycrystalline material with realistic boundary conditions and high spatial and angular resolution. Crystal plasticity simulations of the microscale deformation of instantiations with and without the coherent twin boundary corroborates the experimental characterization and confirms the presence of high gradients in the micromechanical fields, within the GOI, in the vicinity of the coherent twin boundary.

## Results

### Initial characterization and GOI selection

This work studied low solvus, high refractory (LSHR), a nickel-based superalloy containing a well-controlled combination of thirteen elements^25^ and produced via a powder metallurgy consolidation route. Common to nickel-based superalloys, a coherent secondary $\gamma \prime$ precipitate phase is formed during processing, which strengthens the material and is stable at elevated temperatures. The morphology of the microstructure prior to mechanical testing was examined using HEDM, and then the sample was cyclically loaded in order to develop intragranular deformation localization due to plasticity. Following the cyclic loading, the reconstructed HEDM data provided the information to select a GOI, which included a coherent twin, a common site for fatigue crack initiation, and its neighboring microstructure. The GOI was then investigated with DFXM to probe the micromechanical, intragranular information in the vicinity of the coherent twin boundary.

**Fig. 2:**
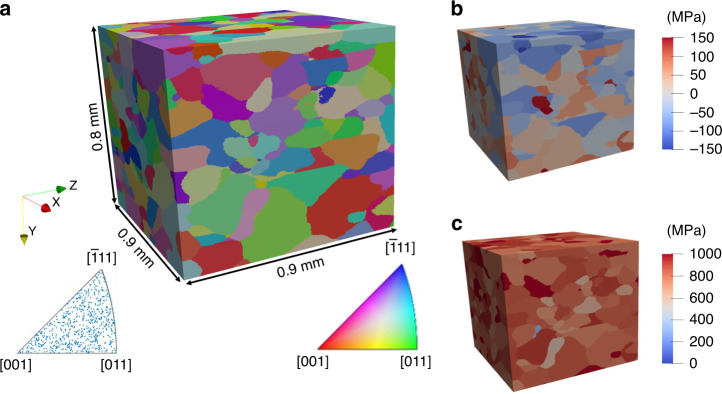
HEDM Characterization. (**a**) Near-field HEDM reconstruction of sample gauge section, colored and described by inverse pole figure (IPF). (**b**) Sample reconstruction colored with grain averaged stresses in the loading direction (y-axis as labeled) when unloaded, prior to loading. (**c**) Sample reconstruction colored with grain averaged stresses in the loading direction when loaded to 1% macroscopic strain, after 1000 cycles.

Near-field HEDM data, acquired at the Cornell High Energy Synchrotron Source (CHESS, beamline F2), were reconstructed to provide grain morphological information, showing that the average grain diameter in the reconstructed microstructure was 65 μm. Additionally, as shown in Fig. 2a, there was little to no observed texture within the material. Following the characterization of the microstructure at the unloaded state, the sample was pre-strained to 1% macroscopic strain and then underwent high cycle fatigue. During the initial loading, the displacement of the load frame was recorded to reach a strain of 1% in the sample. The sample was then cycled, at room temperature, between the maximum and minimum displacement for 1000 cycles (with sequential data collection at 1, 10, and 1000 cycles) (See Supplementary Note 1). Each data collection step involved a far-field HEDM scan to provide the grain-averaged elastic strain for each grain in the loaded microstructure. It can be seen in Fig. 2b that the residual stress distribution, in the initial state prior to cyclic loading, was heterogeneous and contained grains both in tension and compression. Even at a loaded state after 1000 cycles, as shown in Fig. 2c, there is still a heterogeneous distribution of stresses within the material, with grain-averaged stresses both above and below the macroscopic yield of the material. This heterogeneous stress state indicates that each grain will plasticize uniquely and that strain differences between adjacent grains can be exacerbated by loading, potentially accelerating failure. The reconstructed near-field HEDM results in the initial state (which provided orientation and morphological information), as well as the far-field HEDM results (which provided strains/stresses in the microstructure), enabled the selection of the appropriate GOI, and a corresponding set of lattice planes, for further analysis with DFXM.

**Fig. 3:**
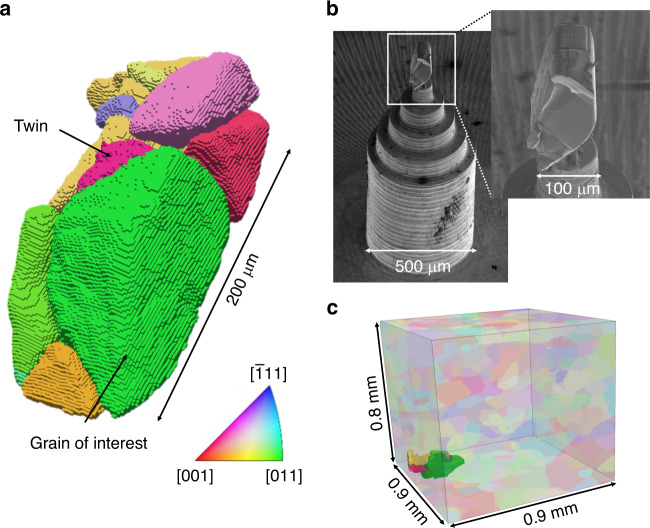
Extraction and DCT Characterization. (**a**) DCT reconstruction of extracted specimen, colored by IPF. (**b**) SEM images of extracted specimen mounted upon a brass pedestal. (**c**) Location of extracted specimen relative to the HEDM reconstruction of the gauge section of the sample.

A GOI was chosen so that its (220) crystallographic planes were correctly oriented with the extraction axis and were likely to exhibit significant lattice curvature due to plasticity localization which was assessed based on diffraction peak broadening as illustrated in Fig. 1a. To maintain the residual stress profiles of the crystallographic planes, a larger microstructural region was extracted around the GOI. Via multiple focused ion beam (FIB) milling techniques and a unique attachment procedure^26^, a roughly cylindrical column with radius of 50 μm and height of 200 μm was extracted from the larger sample (Fig. 3b). The FIB damage is expected to be limited to 50 nm deep into material^27^, which is considered a negligible depth considering the total size of the specimen and the spatial resolution of DFXM (voxel size of 94 nm × 300 nm × 1 μm). DCT, conducted at the European Synchrotron Radiation Facility (ESRF, beamline ID06), provided the 3D morphology and grain orientations of the extracted specimen^28^ (Fig. 3a) which were used to spatially link the DFXM scans to the greater sample reconstructed via HEDM. This spatial linking (Fig. 3c) confirmed that the extraction was successful in capturing a large portion of the GOI, as well as the surrounding microstructure, and provided the ability to match the intragranular information provided by DFXM to the grain-averaged information achieved via HEDM. DCT also captured the presence of a coherent twin boundary (again, a known site for fatigue crack initiation) within the GOI that shared the same (220) planes of interest examined via DFXM.

### Grain of interest study

**Fig. 4:**
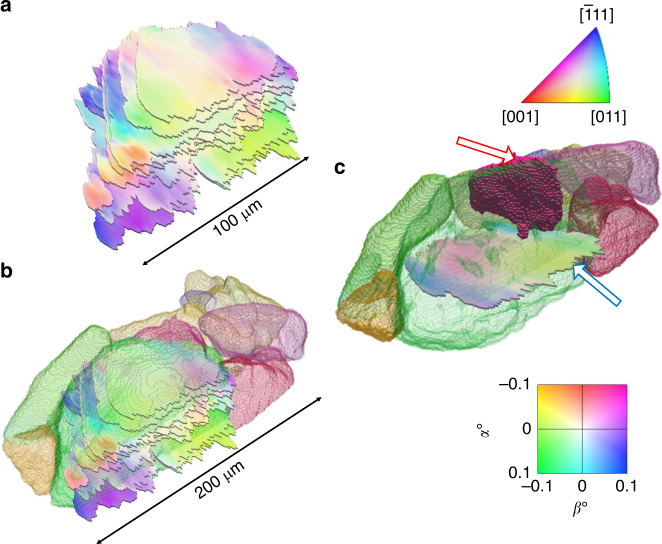
Spatially Linking DCT and DFXM. (**a**) DFXM mosaicity scans, of the GOI, placed within the DCT reconstruction. (**b**) DFXM mosaicity scans, stacked to construct the 3D GOI. (**c**) Spatial location of combined mosaicity and elastic strain scan to be investigated in regards to the coherent twin. Combined mosaicity and elastic strain scan marked is marked by a blue/white arrow while the coherent twin is marked by a red/white arrow.

With two components of lattice plane orientation and one component of elastic strain on the crystallographic planes of interest, DFXM investigated the various microstructural fields of the GOI through an assortment of characterization scans, which each probed a single 1 μm thick layer. The orientation and elastic strain resolutions of this technique, as determined by Poulsen et al., are 0.0057° and 10^−4^, respectively^17^. Conducted at ESRF, beamline ID06, mosaicity (lattice curvature) scans provided 2D spatial information of the lattice curvature. Multiple mosaicity scans taken as the sample was translated vertically were stacked to provide a 3D dataset of the orientation distribution within a single grain after cyclic deformation (Fig. 4a). The mosaicity scans revealed orientation distributions up to 0.3° from one region to another within the GOI. The stacked layers were spatially linked to the DCT reconstruction (Fig. 4b). This spatial registration allowed for the intragranular misorientations to be considered with the knowledge of the surrounding microstructure.

Due to the crystallographic relationship between a parent grain and its twin, some of their crystallographic planes are shared, which results in shared diffraction peaks. The crystallographic set of planes analyzed in this experiment were shared between the parent and twin, and as such, the boundary is not directly visible via DFXM beyond slight lattice curvatures resulting from strain anisotropy between the parent and twin. A coupled mosaicity and elastic strain scan, which simultaneously probed the orientation and elastic strain of the planes of interest, was conducted directly adjacent to the twin boundary mentioned previously (Fig. 4c). The scan showed large amounts of misorientation present in the region near the twin boundary as well as a large tensile strain as compared to the rest of the grain. The misorientation provided from DFXM can be taken as a metric associated with the amount of accumulated plastic strain in the material, hence the coupled mosaicity and elastic strain scan provides key insights into the plastic and elastic strain distributions within the GOI.

**Fig. 5:**
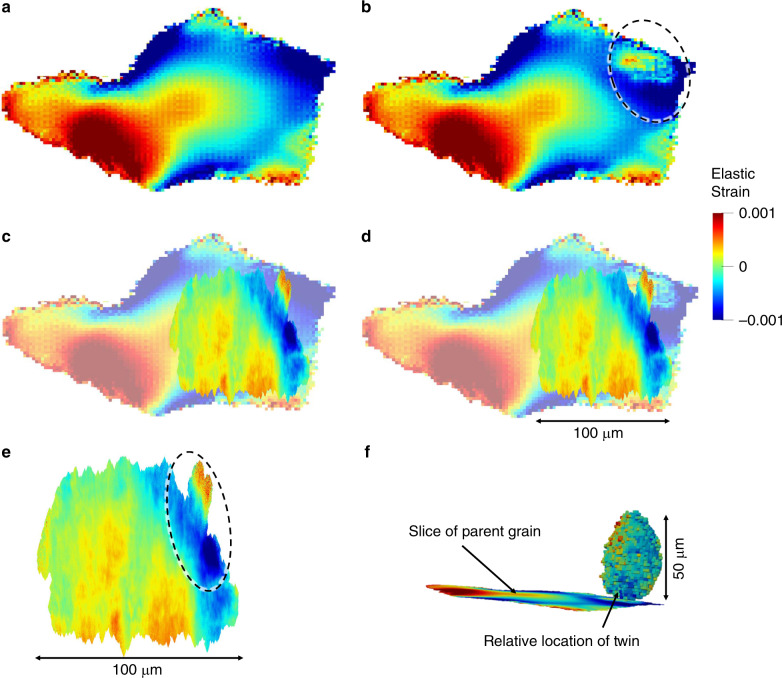
DFXM and CP-FFT Comparison – Elastic Strain. (**a**) CP-FFT model result of elastic strain without twin instantiation; (**b**) with twin instantiation. (**c**) CP-FFT model from a with DFXM overlay from **e**. (**d**) CP-FFT model from **b** with DFXM overlay from **e**. (**e**) Elastic strain from DFXM combined mosaicity and elastic strain scan. (**f**) Relative position of twin boundary to the 2D slices **a**–**e**. All strains are of the elastic strain component normal to the (220) planes of interest.

**Fig. 6:**
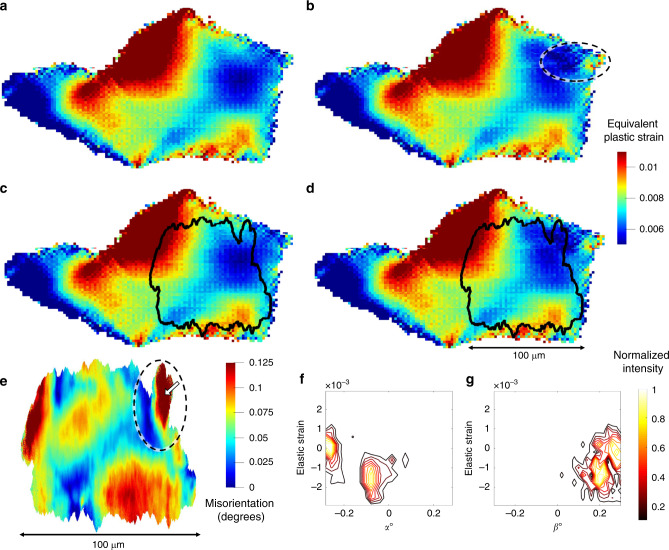
DFXM and CP-FFT Comparison – Plastic Metrics. (**a**) CP-FFT model result of equivalent plastic strain without twin instantiation; (**b**) with twin instantiation. (**c**) CP-FFT model from a with DFXM outline from **e**. (**d**) CP-FFT model from **b** with DFXM outline from **e**. (**e**) Misorientation from DFXM combined mosaicity and elastic strain scan. (**f**, **g**) Contour plots displaying diffraction intensity data from a single voxel within the dipole region of the DFXM combined mosaicity and elastic strain scan labeled by black/white arrow in **e** of elastic strain vs *α* (**f**) and elastic strain vs *β* (**g**).

To compare with the DFXM results, a crystal plasticity model, based on a fast Fourier transform solver, (CP-FFT) was instantiated with the microstructure from the HEDM reconstruction^29–33^. The microstructure was simulated twice, once without the twin boundary instantiated (Figs. 5a, c, 6a, c), and once with (Figs. 5b, d, 6b, d) to directly visualize the effect of the twin boundary on the local micromechanics. To compare the experimental DFXM to the simulated CP-FFT model, the elastic strains extracted from the simulation were projected normal to the (220) planes of interest, allowing for a direct comparison (Fig. 5), while an equivalent von Mises plastic strain was extracted from the model to qualitatively compare to misorientation (Fig. 6). The elastic strains from DFXM exhibit steep gradients in the region in direct proximity to the twin boundary (circled in Fig. 5e). The model confirms the experimentally observed strains when the twin boundary is instantiated (circled in Fig. 5b), whereas, the strain gradient is not present when the twin is absent (Fig. 5c). This indicates that the large strain gradient is due entirely to the microstructural interactions created by the twin boundary (Fig. 5f). Note that while DFXM measures the elastic strain state of a set of planes, the stresses can be estimated using the correct elastic moduli (Supplementary Note 2), and as such, the steep gradients in strain correspond to a residual stress gradient on the (220) crystallographic planes of interest, on the order of 400 MPa over a 30 μm span in the material. The presence of this heterogeneous stress state about the twin boundary, and the confirmation by the CP-FFT model that the micromechanical state is due to the existence of the twin boundary, provides an insight into the micromechanical fields around common fatigue crack nucleation sites in nickel-based superalloys.

As mentioned, lattice curvature is an expected result of plastic deformation localization and as such, the misorientation map acquired from DFXM showed sharp gradients in direct proximity to the twin boundary (circled in Fig. 6e). This sharp change in orientation is likely due to the presence of a high dislocation density^34^, specifically geometrically necessary dislocations (GNDs); GNDs have been shown to accumulate at grain and sub-grain boundaries^19^. Similarly, the CP-FFT model displayed a gradient feature in the equivalent von Mises plastic strain, but only when the twin boundary was instantiated (circled in Fig. 6b). An exact spatial match is not expected between the simulation and DFXM as the grain boundaries modeled in the CP-FFT simulation were reconstructed from NF-HEDM, which has a lower spatial resolution than DFXM. Nevertheless, there are common trends that exist between the two, such as the diagonal path of lower plastic strain across the grain and the region of high plastic strain in the right lower corner of the GOI (Fig. 6d). The location circled in Fig. 6e showed an extreme deviation from the rest of the GOI in misorientation (again a metric of plastic deformation) resulting in a lower diffraction intensity within the tilt range chosen for the scan. This low diffraction intensity near the twin boundary was likely due to large distortions in the lattice, which typically result from high dislocation densities caused by large amounts of accumulated plastic strain. It was also observed that multiple orientations exist within a single voxel in the region directly influenced by the twin boundary (Fig. 6f, g). The contour plots, shown in Fig. 6f, g, illustrate this dual orientation with multiple distinct peaks within a single voxel, of volume 0.0282 μm^3^, each holding an orientation and elastic strain separated by 0.2° and 0.0015, respectively. This elastic strain difference corresponds to a 300 MPa residual stress separation, similar in magnitude to those seen in Levine et al.^22^. Such a difference could be explained by the $\gamma \prime$ precipitates within the material becoming decoherent and now, in a residual state, holding a different orientation and stress value than the surrounding *γ* matrix. The dual orientation could also be explained by a very high dislocation content creating a cell structure, where the cell walls hold a different orientation than the interior matrices^35^.

Our analysis to this point has investigated the distinct quantities of elastic strains (thereby stress) and misorientation (as a surrogate to equivalent plastic strain), as there has been extensive work done showing that both stress concentrations and strain localizations (in the form of high GND density and local slip activity) are present at fatigue crack nucleation sites, such as coherent twin boundaries. While our work does not bring the sample to failure, it distinctly shows that both elastic strain (stress) and misorientation (a metric of plastic strain) display localized gradients in a region well known for fatigue crack initiation, supporting the need to investigate both terms simultaneously. Figures 5 and 6 both also show that coherent twin boundaries are not the only sites prone to damage accumulation. The left side of the GOI, as shown by the CP-FFT model, contains regions with no twin boundaries but similar micromechanical field gradients; however, multiple arbitrary boundaries lie, both in plane and out of plane, of the figures shown. This left side region, though illuminated during DFXM scanning, did not diffract within the tilt ranges of the combined mosaicity and elastic strain scan, implying that the orientation of this region was misorientated outside of the range displayed in Fig. 6e. This misorientation is reflected also in the CP-FFT results as the region to the left of the DFXM boundary seen in Fig. 6d displays large regions of high and low equivalent plastic strain. Both regions also display the need for 3D analysis in future research activities to achieve a robust deterministic failure criterion. However, while 2D characterization often provides simplified analyses, 3D characterization often has the opposite effect, creating datasets too vast to analyze without conducting lower dimensional analysis^36^. Therefore, the need arises for a balance of 2D characterizations and models to first present a theory, then an extensive 3D characterization and model to fully capture microstructural elements, such as a twin boundary in the bulk of a sample surrounded by a complex microstructure, to further confirm theories produced by the 2D characterization.

## Discussion

The importance of twin boundaries on the fatigue performance of nickel-based superalloys has been well documented due to their ability to increase strength, by impeding dislocation motion, while maintaining the overall ductility of the material. By impeding dislocation motion however, strain is localized along the coherent twin boundary, and as this strain localization increases, an incompatibility begins to form on either side of the twin boundary, generating a large stress concentration. As shown via surface techniques, the stresses become too large for the material to accommodate and a fatigue crack initiates^37,38^. However, a deterministic failure criteria, which by necessity would involve the sub-surface, 3D microstructure (where many failures occur, especially on surface treated components) is not sufficiently understood. The experimental data shown here display that the sub-surface region of the parent grain, in direct proximity to the twin boundary, exhibits sharp gradients in both stress and strain in an attempt to maintain compatibility with its smaller twin. The experimental characterization and the CP-FFT model confirm that the twin boundary is the direct cause of these large gradients. These results provide insight into the development of the micromechanical fields prior to fatigue crack initiation and into the metrics, such as stress and strain gradients, which could help define a fatigue failure criteria.

These results elucidate the need for characterizations with high spatial and angular resolution to capture the sharp gradients of intragranular strain that exist near grain boundaries. The present study provides a protocol for similar multi-scale experiments that can be conducted completely in situ with future planned upgrades to existing synchrotron sources. Further, the shown sub-surface strain results are unable to be captured by techniques such as TEM, which relieve any residual strains that exist due to the small sample size, nor HR-EBSD as it only captures surface deformation. This work illustrates the power of combining a uniquely tailored multi-scale experiment with a crystal plasticity model and shows the need for similar combinations to be used to zoom-in on sites which are susceptible to failure. Additionally, nickel-based superalloys are only one of the many material systems which are used for critical, structural components, and are prone to other failure mechanisms at sites such as inclusions, precipitates, and unique grain boundary types or clusters. Many of these have not yet been investigated at a length scale necessary to elucidate the physics governing their failure. Sub-surface, high-resolution characterizations identifying and quantifying high stress gradients, as shown here, provide critical information necessary to develop micromechanical rationale for failure mechanisms such as fatigue crack initiation. As such, future work is needed for the development and validation of the next generation of advanced, multi-scale, mechanical performance models, which will lead to more accurate lifetime prediction of structural components with reduced uncertainty.

## Methods

### Material description

The material used throughout the experiments was LSHR (low solvus, high refractory) and its composition, along with general properties, are detailed by Gabb et al.^25^. The material was isothermally forged and then underwent a 1 h supersolvus heating at 1171 °C. The material was aged at 855 °C for 4 h, followed by 775 °C for 8 h, and cooled in air. The sample was manufactured via electrical discharge machining to the final, millimeter sized sample, shown in Fig. 1a.

### HEDM characterization and fatigue experiment

Initial characterization of the sample was performed at the CHESS, beamline F2, via HEDM. HEDM is an X-ray technique that rotates a millimeter sized sample, while illuminated by a monochromatic X-ray beam, where the multiple modalities of HEDM can be used to reconstruct the morphology of the grains, along with the 3D grain-averaged lattice strain tensor for each grain in the illuminated region^39–41^ (Fig. 1a). Far-field HEDM (FF-HEDM) uses area detectors placed roughly a meter downstream from the sample to capture the diffraction peaks from the rotating polycrystal. With this large distance between the sample and the detector, the location of the peak upon the detector is dominated by the angle at which the incident X-rays are diffracted, providing a resolution in orientation measurements of ±0.01° and elastic strain of ±1 × 10^−4^ at CHESS^42^. HEDM analysis was conducted with a monochromatic X-ray beam energy of 61.332 *keV*. FF-HEDM was conducted with a detector distance of 734 mm via two Dexela 2923 area detectors with 3888 × 3072 pixels each, with a pixel size of 74.8 μm. The FF-HEDM was conducted on a 1 mm tall region with ten scans of beam size 120 μm × 2.5 mm and was reconstructed using the HEXRD software^40^.

A complementary technique, near-field HEDM (NF-HEDM), uses a detector placed millimeters from the sample. At this distance, the locations of the diffraction peaks upon the area detector are informed by the spatial locations of diffracting crystal volumes, and thus provide a lower angular resolution but much higher spatial resolution (<0.1° and 2 μm, respectively^43,44^). The NF-HEDM was conducted with a detector distance of 6.40 mm using a LuAg:Ce scintillator paired with a 5× objective lens to a Retiga 4000DC CCD camera which resulted in 2048 × 2048 pixels with an effective pixel size of 1.48 μm. NF-HEDM was conducted on an 800 μm tall region with eight diffraction volumes of size 120 μm × 2.5 mm and analysis was done by a seeded forward-modeling based reconstruction method^41,45^ with FF-HEDM data and used a voxel spacing of 2 μm. Three gold cubes were placed upon the sample’s surface^26^ prior to the HEDM experiment, and subsequent cyclic loading, for spatial registration of the near and far-field diffraction volumes.

The cyclic loading was facilitated by the RAMS2 load frame^46^, and the loading sequence was chosen to induce sufficient plasticity within the polycrystalline aggregate for DFXM to resolve phenomena such as strain localization at grain boundaries. To track the evolution of the individual grains due to the cyclic loading, FF-HEDM was conducted to determine the grain-averaged elastic strains (and thus stresses with knowledge of the single-crystal elastic moduli) sequentially at: just under and over the macroscopic yield of the material, and while holding peak load at 1, 10, and 1000 cycles. The reconstructed data from both NF-HEDM and FF-HEDM were combined and post processed via a combination of an in-house Matlab script and a Dream3D pipeline, then visualized in ParaView.

### Specimen preparation

The size limitation of DFXM, when taking into account the 33 keV energy of the monochromatic X-ray beam, required unique extraction techniques to physically remove a GOI and its surrounding microstructure from the larger sample. To link the sub-surface GOI’s location between the NF-HEDM reconstruction and the larger sample, EBSD characterization was conducted to inform the exact position for extraction (Supplementary Note 3). Initial spatial marking was completed at the Air Force Research Laboratory (AFRL) via a liquid metal ion source FIB (specifically Ga). Large volume material removal around the columns to be extracted was done via plasma-FIB at the NASA Langley Research Center. Final extraction of the column and pedestal attachment was completed at AFRL (Supplementary Note 4). The column was extracted from the larger sample using a micromanipulator and mounted to a pre-cut brass pedestal via platinum deposition^26^.

### DCT characterization

The DCT analysis was conducted at ESRF, beamline ID06, with a beam energy of 33 keV on a scintillator screen connected via microscope optics to a FReLoN CCD camera resulting in an effective pixel size of 1.24 μm. The detector was placed 6 mm from the specimen, laterally offset from the beam axis by 1.15 mm. In this position, diffraction spots of the innermost five *hkl* families could be captured while continuously rotating the sample over 360° in steps of 0.1°. Following the processing route described in Reischig et al. and Vigano et al.^47,48^, a total of 24 grains, including a series of smaller annealing twins could be indexed and reconstructed from this acquisition. A comparison of the specimen’s microstructure from the DCT reconstruction and the larger sample’s microstructure from NF-HEDM is provided in Supplementary Note 5. A reference frame correction between the HEDM characterization at CHESS and the DCT volume obtained at ESRF was determined via an optimization scheme.

### DFXM Characterization

DFXM was conducted at ESRF, beamline ID06, and utilized a similar microscopy detector composed of a scintillator, microscope with 10× objective, and FReLoN CCD camera which resulted in a 1.4 μm pixel size. In addition, a SU-8 resist compound refractive lens, made up of 65 lens elements, was placed in the diffracted beam, 335 mm downstream of the specimen and 4967 mm upstream of the microscopy detector, to provide an X-ray magnification of 14.82. Upstream of with the sample, the incoming X-ray beam was focused into a line of 1 μm via a set of linear compound refractive lenses. With the magnification, detector system, and experimental setup, the voxel size is 94 nm × 300 nm × 1 μm. In angular space, DFXM has been shown to obtain orientation and strain resolutions of 0.0057° and 10^−4^, respectively^17^. Further information on the resolutions, experimental setup, and methodology are described by Poulsen et al. and Simons et al.^17,18^.

The scanning procedure for the mosaicity scans rocked the two tilts *α* and *β* with 0.02° steps over a range of 0.4° and 0.6°, respectively. The coupled mosaicity and elastic strain scans rocked both *α* and *β* while also tilting the objective lens. The tilts *α* and *β* were both rocked over a range of 0.6° with step sizes of 0.03° and 0.02°, respectively. The objective was tilted a total of 0.1° over 15 steps. To maintain the interrogation region within the specimen and the image location upon the detector, the objective tilt was coupled with a vertical objective translation of 0.5595 mm over the 15 steps. This resulted in a mosaicity scan that mapped the lattice curvature of the illuminated planes, and as the specimen was tilted through the scan ranges, parts of the grain passed in and out of the diffraction condition. This generated a 2D mesh of intensity values for each voxel of material within the GOI across the *α* and *β* ranges. The centroid of this mesh was found with a center-of-mass fit and orientations based on the *α *and *β* values were assigned to each voxel to produce mosaicity maps. Additionally, the orientations from the *α* and *β* rotations were combined via a sum of squares to generate a map displaying a metric of total misorientation. Coupled mosaicity and elastic strain scans concurrently mapped a degree of freedom in strain space, 2*θ* (the elastic strain component normal to the planes of interest), and two orientation degrees of freedom, *α* and *β*, to link elastic strain values with the orientation of the voxel. From objective tilt, elastic strain is calculated as

1 $${\it{\epsilon }} = - \frac{1}{2}\frac{{{\mathrm{\Delta }}2\theta }}{{{\rm{Tan}}\left( {\frac{{2\theta }}{2}} \right)}},$$

where 2*θ* is the angle satisfying the Laue condition for the crystallographic planes of interest and Δ2*θ* is the objective tilt which selects small deviations from 2*θ*. This coupled scan was done by completing mosaicity scans across a range of objective tilts to create a 3D volume in orientation/strain space for each voxel, allowing the determination of both mosaicity and elastic strain, simultaneously. Each DFXM scan was of a 1 μm thick layer of the GOI. Due to the experimental setup, the vertical translation step size was not stable which required a post experiment, manual shift of the images in detector space to correct for the image translation upon the detector. Data analysis was completed via an in-house Matlab script adapted from those used in Simons et al.^18^.

The experimental setup had limits on the maximum tilt the specimen can achieve, which required the crystallographic planes of interest to be pre-aligned during the GOI extraction. To capture the dislocation mechanics of the nickel-based superalloy studied in this work (FCC crystal structure), either the (111) or (220) families of planes could be selected for investigation, which is based on the dislocation slip mechanics of FCC crystals. In addition, ample plastic deformation accommodated by the crystallographic planes was desired for proper investigation of the deformation mechanics after cyclic loading. While current implementations of FF-HEDM use the indexed centroids of the diffraction peaks on the detector to determine grain average properties, the shape of the peak also provides useful information of the misoriented state of the grain. The intragranular misorientation and lattice strain gradient information provided by the peak shape are indicative of plastic deformation processes, where highly misoriented or strained regions of a grain will skew its diffraction peaks from their original distributions^49^. Therefore, via the grain-averaged orientation information provided by FF-HEDM, multiple candidate grains were chosen, and then an in-house reciprocal space mapping technique^50^ was used to determine a metric of the total amount of plastic deformation within each candidate grain.

The spatial alignment between the two X-ray techniques was not exact; the boundary of the DFXM scans are defined by thresholds dictated by median filters and are subject to errors arising from X-ray effects and microscope out-of-focus blurring, though calibration routines at the start of data acquisition help reduce the effect of the latter. Additionally, during acquisition, a set range of motor tilts were chosen to balance the acquisition of the entire orientation space defined by the GOI and the acquisition time. These requirements caused regions, such as those of significant misorientation or high dislocation density content, to never come fully into the diffraction condition, which resulted in spatial regions with little to no intensity. However, with the information of the surrounding microstructure from DCT, these heavily misoriented regions were often linked to grain boundaries. The regions of high misorientation near grain boundaries indicate deformation processes such as lattice rotation and dislocation pile up.

### CP-FFT modeling

The CP-FFT model was informed with the 3D microstructure reconstructed from NF-HEDM (with the same spatial resolution of 2 μm per voxel) and grain-averaged orientations determined via FF-HEDM. The NF-HEDM reconstruction was used to make two instantiations of the CP-FFT model: one instantiation without the twin, and one instantiation with the twin’s morphology, which was created by the insertion of voxels that held a minimum confidence of 80% for the twin’s orientation. The NF-HEDM reconstruction used in the simulation without the twin instantiated was not seeded with the orientation of the twin. The CP-FFT model enforced the macroscopic strain rate along the loading direction defined during sample cyclic loading, no other boundary conditions were prescribed. The model was run to strain the volume to 1% macroscopic strain then unloaded to 0 N, identical to the initial experimental loading. The model was run for a single cycle where it was determined that the computation time of further cycling was not necessary as a good match was found between the micromechanical fields from DFXM and CP-FFT, which were not expected to evolve qualitatively due to the form of the constitutive equations used^51^. Due to the Fourier transform formulation of the model, the volume had to have periodic boundary conditions. To ensure continuity to transmit load, the microstructure was mirrored along the loading direction; the artificial mirrored boundary did not affect the results of this study, as multiple grains lay between the artificial boundary and our GOI^33^. Along the other directions, a gas phase of zero stiffness was added to simulate the free surfaces. The model’s constitutive relationship used to describe the material’s hardening behavior was the generalized Voce law^52^. The governing equations (Supplementary Note 6) and simulation routine are described further by Lebensohn et al.^32^. The Voce hardening and crystal plasticity parameters were fit by calibrating the macroscopic stress-strain curve of the model to the experimentally captured curve. The parameters fit for both models, with and without the twin instantiated, were $\tau _0 = 277.75\,{\rm{MPa}},\tau _1 = 3.0\,{\rm{MPa}},\theta _0 = 19519.5\,{\rm{MPa}},\theta _1 = 210.0\,{\rm{MPa}},n = 12.0,\dot \gamma _0 = 0.056$. The necessary values of the stiffness tensor were taken from Cerrone et al.^53^. To match the experimental load state of DFXM, the CP-FFT model results shown in Figs. 5 and 6 are in the unloaded state, after being cycled once, to 1% macroscopic strain.

## Supplementary information

Supplementary Information

## Data Availability

The datasets generated and then analyzed during the current study are available upon reasonable request to the corresponding author.

## Code availability

The HEDM datasets were reconstructed via the X-ray diffraction analysis software, HEXRD, which is available on https://github.com/HEXRD.
