# Supplementary material for: Quantifying microscale drivers for fatigue failure via coupled synchrotron X-ray characterization and simulations
Source: Nat Commun. 2020 Jun 24;11:3189. doi: 10.1038/s41467-020-16894-2 (PMC7314802; doi:10.1038/s41467-020-16894-2)
Supplement: Supplementary file 1 — Supplementary Information [file 41467_2020_16894_MOESM1_ESM.pdf]

Quantifying microscale drivers for fatigue failure via coupled synchrotron X-ray characterization and simulations

Gustafson et al.

Supplementary Notes:

Supplementary Note 1:

Supplementary Figure 1 describes the macroscopic loading curve captured by digital image correlation during mechanical loading at CHESS. FF-HEDM scans as described by the figure below were all taken of a $800\mu m$ length of the gauge section. Only one cycle was captured via this measurement process to speed the cyclic testing.

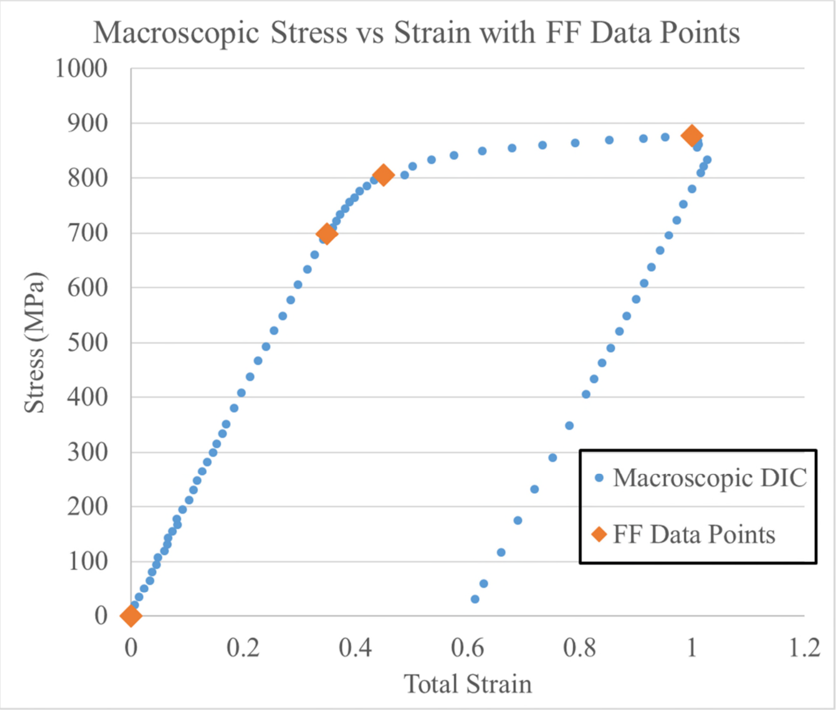

Supplementary Figure 1: Macroscopic loading curve for HEDM sample

Supplementary Note 2:

Given the FCC crystal structure, general elasticity theory can be applied to estimate the elastic modulus for a set of crystallographic planes via Equation 1.

$$E = \frac{1}{S_{11} - 2\left(S_{11} - S_{12} - \frac{1}{2}S_{44}\right)(\alpha^2\beta^2 + \alpha^2\gamma^2 + \beta^2\gamma^2)} \tag{1}$$

Where $\alpha, \beta, and\ \gamma$ are the normalized dot products of the plane's normal vector and the basis vectors and $S_{11}, S_{12}, and\ S_{44}$ are the compliance tensor components determined from the stiffness tensor components via Equations 2-4.

$$S_{11} = \frac{C_{11} + C_{12}}{C_{11}^2 + C_{11}C_{12} - 2C_{12}^2}; S_{12} = \frac{C_{12}}{-C_{11}^2 - C_{11}C_{12} + 2C_{12}^2}; S_{44} = \frac{1}{C_{44}} \tag{2-4}$$

The stiffness tensors component values from Cerrone el al.[1] (in GPa) are $C_{11} = 238.5, C_{12} = 148.4, and\ C_{44} = 123.5$. This resulted in an elastic modulus of 222.4 GPa on the (220) crystallographic planes of interest.

Supplementary Note 3:

Once a candidate grain was chosen so that its crystallographic planes of interest were aligned well to the extraction axis (their normal was perpendicular to the normal of the surface shown in Supplementary Figure 1), the grain had to be extracted from the sample. The grain of interest (GOI) chosen was subsurface, as show in Supplementary Figure 2c, and thus to minimize sources of uncertainty, an electron backscatter diffraction (EBSD) image was taken of the surface to compare to the reconstructed surface from near field high energy X-ray diffraction microscopy (NF-HEDM). The EBSD scan was performed on a Phillips XL30 scanning electron microscope with an accelerating voltage of 20 keV. The large area EBSD scan shown in Supplementary Figure 2b consists of an array of scans each with size 270 $\mu m \times 270$ μm with 24 μm of overlap between scans. The 30 separate tiles (5×6) were stitched together[2,3]. As shown in Supplementary Figure 2b the microstructure reconstructed via NF-HEDM compares well with the EBSD scan. Via visualization in ParaView, the surface location of extraction was determined and is shown by the black boxes in Supplementary Figure 2a and 2b. Note that the EBSD scan was taken after a polishing routine and as such, its depth into the sample was unknown, which affects the spatial match of the two techniques.

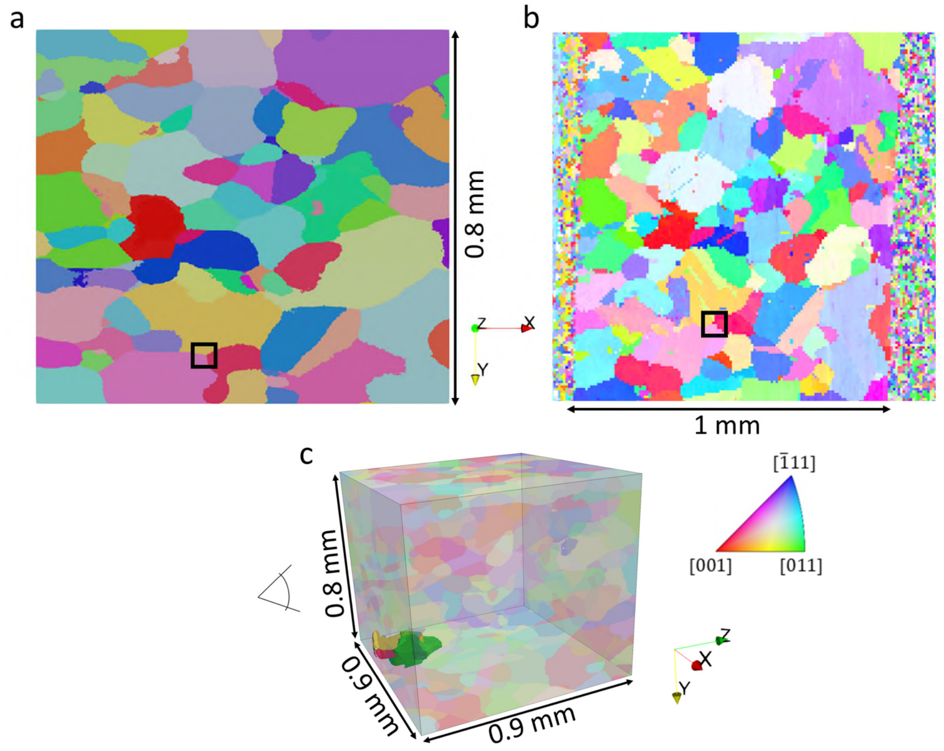

Supplementary Figure 2: a) slice of NF-HEDM reconstruction at the surface, b) EBSD scan, and c) location of the GOI relative to larger sample with a viewpoint for the EBSD scan.

Supplementary Note 4:

A total of three grains were identified as possible candidates for extraction. As shown in Supplementary Figure 3a, there are three squares cut into the material (the particular GOI's square is outlined in red) marking the subsurface location of the grain.

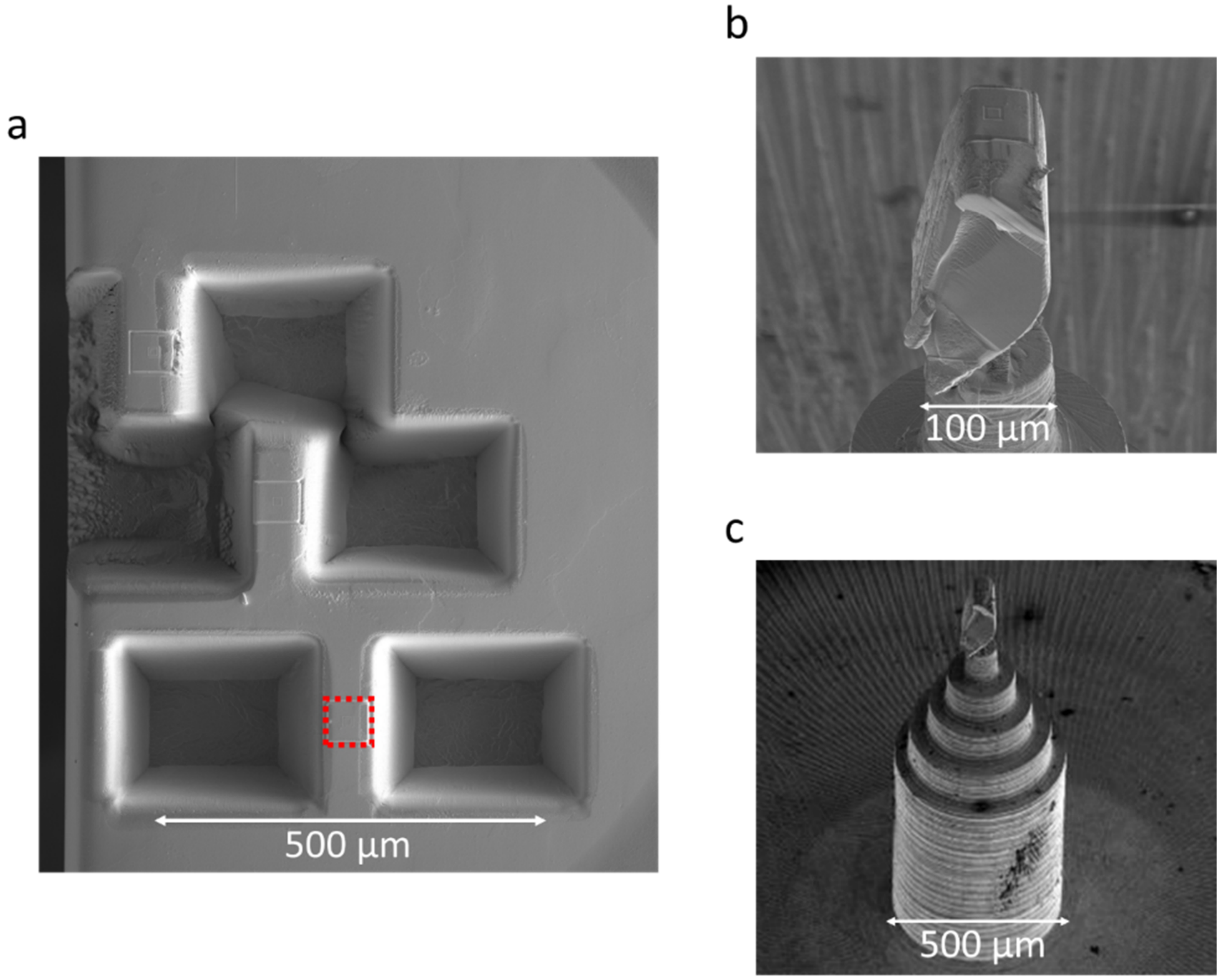

Supplementary Figure 3: a) Sample surface with extraction locations marked, after the large material removal via plasma-FIB, b) zoomed image of the extracted specimen, and c) image of the extracted sample mounted atop a brass pedestal.

Supplementary Note 5:

In order to link the fatigue loading data collected from far-field HEDM (FF-HEDM), the morphology of the extracted specimen had to be linked to the larger sample. The larger sample's microstructure was reconstructed from NF-HEDM, while the smaller specimen's microstructure was reconstructed from diffraction contrast tomography (DCT). Prior to spatial linking, a reference frame correction had to be applied to the orientations found from DCT. The reference frame correction was determined via an in-house, Matlab optimization routine to transform the orientations in the ESRF frame to the CHESS frame. As multiple grains could be spatially matched between the two data sets, the misorientations between the orientations assigned to the same grain by the two different techniques could be found. The optimization applied a random rotation (full rotation comprising of three Euler angles) to the ESRF orientations, then adjusted the rotation to minimize the total (summed) misorientation difference between known pairs of grains. After multiple iterations, a good match was found between the data sets where the summed misorientation from four known grain pairs was 1.5°. To spatially compare NF-HEDM to DCT, slices of NF-HEDM were cut where they intersected with the DCT volume and is shown at two perspectives in Supplementary Figure 4a,b. The DCT volume was then replaced with only its outline at the plane of intersection between NF-HEDM and DCT in Supplementary Figure 4c,d. Supplementary Figure 4 displays a good match between NF-HEDM and DCT with only slight morphological differences likely due to the detector resolution differences between the two techniques.

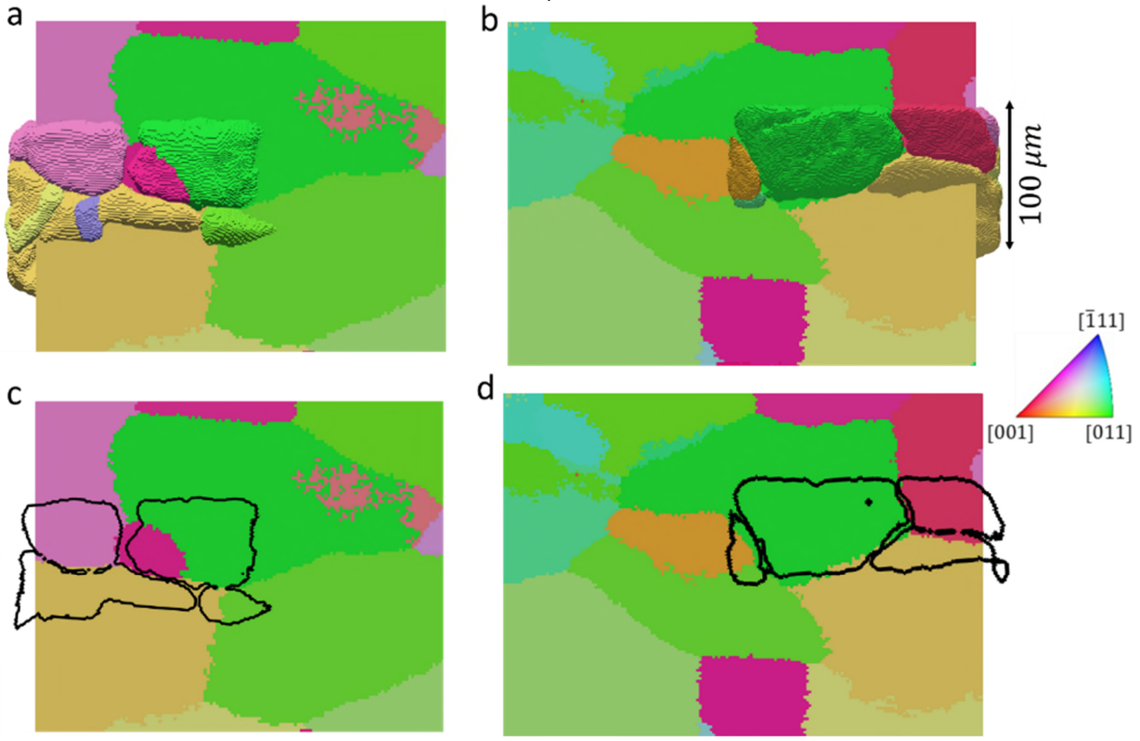

Supplementary Figure 4: a and b) DCT reconstruction sliced by NF-HEDM reconstruction. c and d) outline of DCT reconstruction on NF-HEDM reconstruction

The images provided in Supplementary Figure 5 show orthogonal and 3D views of the extracted microstructure reconstructed from DCT to better demonstrate the physical location of the GOI and its relationship to the gauge section as a whole.

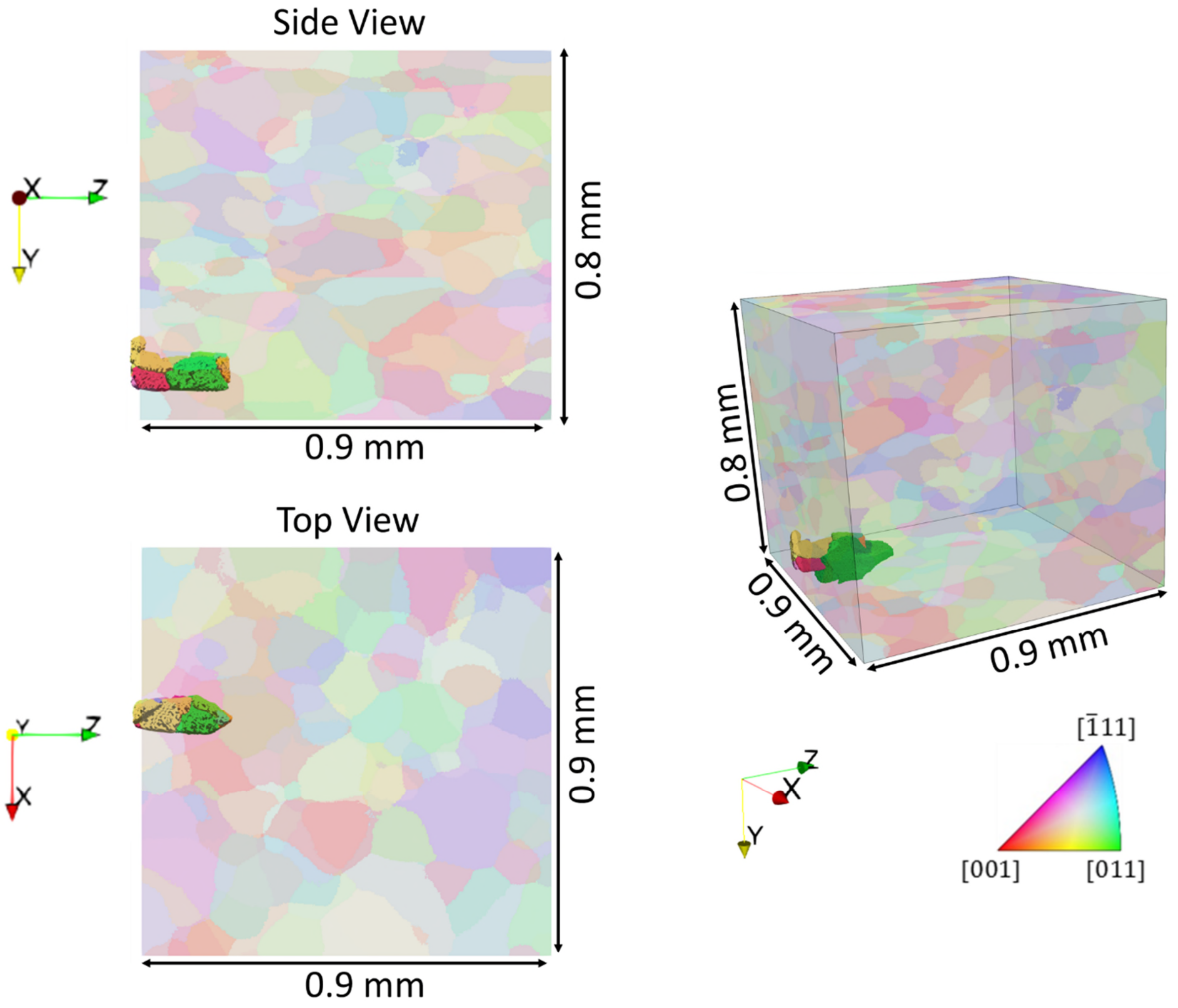

Supplementary Figure 5: Orthogonal views displaying the relative size and position of the region of interest (and GOI) within the gauge section of the HEDM sample.

Supplementary Note 6:

The crystal plasticity model used to simulate the loading conditions applied to the HEDM sample employed an elasto viscoplastic Fast Fourier Transform method[4,5]. This modeling effort was used to rationalize the intragranular micromechanical fields to compare to the dark field X-ray microscopy experimental data. This model has been shown to be reliable after a comparison to experiments and finite element based approaches and has been used on a number of different material systems[6–8]. This model calculates a compatible strain field and an equilibrium stress field for each point (x) within the microstructure. The elastic relation, in the crystal frame, at time $t + \Delta t$ is described in Equation (5) as:

$$\begin{aligned}\sigma(x) &= C(x): \epsilon^{el}(x) \\ &= C(x): \left(\epsilon(x) - \epsilon^{pl}(x)\right) \\ &= C(x): (\epsilon(x) - \epsilon^{pl,t}(x) - \dot{\epsilon}^{pl}\Delta t)\end{aligned} \quad (5)$$

where $C(x)$ is the fourth-order stiffness tensor, $\sigma(x)$ is the stress tensor at point (x), $\epsilon^{pl,t}$ is the plastic strain at time t, and $\dot{\epsilon}^{pl}$ is the plastic strain rate at time $t + \Delta t$. The plastic strain rate, at time t, is described in Equation (6) as:

$$\dot{\epsilon}^{pl}(x) = \sum_{\alpha=1}^{N} M^{\alpha}(x)\dot{\gamma}^{\alpha}(x) = \dot{\gamma}_0 \sum_{\alpha=1}^{N} M^{\alpha}(x) \left(\frac{|M^{\alpha}(x): \sigma(x)|}{\tau_{CRSS}(x)}\right)^{n} sgn\left(M^{\alpha}(x): \sigma(x)\right) \quad (6)$$

Here, N is the number of slip systems (which for FCC crystals such as nickel have 12), $M^{\alpha}(x)$ is the symmetric Schmid tensor for the slip system α, and $\dot{\gamma}^{\alpha}$ is the resolved shear strain rate on system α. Also, $\dot{\gamma}_0$ is the shear strain normalization factor, n is the rate sensitivity exponent, and τ_{CRSS} is the critical resolved shear stress. The resolved shear stress is related to the stress tensor as described in Equation (7):

$$\tau^{\alpha}(x) = M^{\alpha}(x): \sigma(x) = \frac{1}{2}\left(m^{\alpha}(x) \otimes n^{\alpha}(x) + n^{\alpha}(x) \otimes m^{\alpha}(x)\right): \sigma(x) \quad (7)$$

where n^{α} is the normal to the slip plane and m^{α} is the slip direction. The microstructure was modeled as a single phase with hardening relationship described by the Voce law as described by Equation (8)[9]:

$$\tau(\Gamma) = \tau_0 + (\tau_1 + \theta_1\Gamma)\left[1 - e^{-\frac{\Gamma\theta_0}{\tau_1}}\right] \quad (8)$$

where Γ is the accumulated plastic shear strain at each point x and defines the value of τ_{CRSS} used in Equation 6. τ_0 and θ_0 are the initial yield stress and hardening rate while τ_1 and θ_1 describe the asymptotic behavior of the stress/strain curve.

Supplementary References:

1. Cerrone, A. *et al.* Implementation and verification of a microstructure-based capability for modeling microcrack nucleation in LSHR at room temperature. *Model. Simul. Mater. Sci. Eng.* **23**, 035006 (2015).
2. Pilchak, A. L., Shiveley, A. R., Tiley, J. S. & Ballard, D. L. AnyStitch: A tool for combining electron backscatter diffraction data sets. *J. Microsc.* **244**, 38–44 (2011).
3. Shiveley, A. R., Shade, P. A., Pilchak, A. L., Tiley, J. S. & Kerns, R. A novel method for acquiring large-scale automated scanning electron microscope data. *J. Microsc.* **244**, 181–186 (2011).
4. Lebensohn, R. A., Kanjarla, A. K. & Eisenlohr, P. An elasto-viscoplastic formulation based on fast Fourier transforms for the prediction of micromechanical fields in polycrystalline materials. *Int. J. Plast.* **32**–**33**, 59–69 (2012).
5. Moulinec, H. & Suquet, P. A numerical method for computing the overall response of nonlinear composites with complex microstructure. *Comput. Methods Appl. Mech. Eng.* **157**, 69–94 (1998).
6. Prakash, A. & Lebensohn, R. A. Simulation of micromechanical behavior of polycrystals: Finite elements versus fast Fourier transforms. *Model. Simul. Mater. Sci. Eng.* **17**, (2009).
7. Rovinelli, A., Proudhon, H., Lebensohn, R. A. & Sangid, M. D. Assessing the reliability of fast Fourier transform-based crystal plasticity simulations of a polycrystalline material near a crack tip. *Int. J. Solids Struct.* **184**, 153–166 (2020).
8. Naragani, D. *et al.* Investigation of fatigue crack initiation from a non-metallic inclusion via high energy x-ray diffraction microscopy. *Acta Mater.* **137**, 71–84 (2017).
9. Tome, C., Canova, G. R., Kocks, U. F., Christodoulou, N. & Jonas, J. J. The relation between macroscopic and microscopic strain hardening in F.C.C. polycrystals. *Acta Metall.* **32**, 1637–1653 (1984).
